# Targeting Redox Homeostasis with Arvanil Is Preferentially Cytotoxic to Glioma Stem Cells

**DOI:** 10.3390/ijms27209358

**Published:** 2026-10-21

**Authors:** Kazuki Nakamura, Senri Takenouchi, Yurika Nakagawa-Saito, Yasufumi Ito, Yuta Mitobe, Shuhei Suzuki, Keita Togashi, Asuka Sugai, Yukihiko Sonoda, Chifumi Kitanaka, Masashi Okada

**Affiliations:** 1 Department of Molecular Cancer Science, Yamagata University School of Medicine, 2-2-2 Iida-nishi, Yamagata 990-9585, Japan; 2 Department of Neurosurgery, Yamagata University School of Medicine, 2-2-2 Iida-nishi, Yamagata 990-9585, Japan; 3 Department of Obstetrics and Gynecology, Yamagata University School of Medicine, 2-2-2 Iida-nishi, Yamagata 990-9585, Japan; 4 Department of Clinical Oncology, Yamagata Prefectural Shinjo Hospital, 720-1 Kanazawa, Shinjo 996-8585, Japan; 5 Department of Ophthalmology and Visual Sciences, Yamagata University School of Medicine, 2-2-2 Iida-nishi, Yamagata 990-9585, Japan

**Keywords:** glioblastoma multiforme, tumor-initiating cells, redox homeostasis, calcium, reactive oxygen species

## Abstract

Glioma stem cells (GSCs) are cancer stem cells in glioblastoma (GBM) that are resistant to both radiotherapy and chemotherapy and contribute to tumor recurrence. Therefore, the development of GSC-targeted therapies is urgently needed to improve the poor prognosis of GBM patients. Given the growing interest in redox homeostasis, which is closely linked to mitochondrial metabolism supporting the unique properties of GSCs, we focused on capsaicin, a widely used exogenous calcium channel agonist, and its structural analogs as candidate compounds capable of disrupting redox homeostasis in GSCs. Based on a comprehensive evaluation of the effects of the candidate compounds, we found that the capsaicin analog arvanil selectively induced cell death in GSCs with high selectivity over differentiated GSCs at concentrations that were non-toxic to normal cells. Arvanil-induced cell death was associated with a preferential elevation in intracellular reactive oxygen species (ROS) levels in GSCs over those in differentiated GSCs and was suppressed by a Ca^2+^ chelator and a mitochondria-targeted antioxidant. These results suggest that arvanil preferentially induced GSC death in association with intracellular Ca^2+^ dysregulation and mitochondria-related oxidative stress, highlighting potential vulnerabilities in the redox homeostasis of GSCs and supporting further investigations on arvanil as a feasible therapeutic approach for GBM.

## 1. Introduction

Glioblastoma (GBM) is the most common primary malignant brain tumor in adults and remains one of the most lethal human malignancies. The current standard treatment consists of maximal safe surgical resection followed by radiotherapy combined with temozolomide-based chemotherapy. Despite these multimodal treatments, most patients eventually experience tumor recurrence, and the long-term prognosis remains poor [1,2]. Cancer stem cells (CSCs) comprise a small subpopulation of tumor cells characterized by their tumor-initiating capacity and resistance to conventional therapies. Due to these properties, CSCs may survive initial treatment and subsequently drive both local and distant tumor recurrence, thereby contributing to poor long-term clinical outcomes [3,4,5]. In GBM, a CSC-like population known as glioma stem cells (GSCs) has been identified. GSCs exhibit marked resistance to radiotherapy and chemotherapy and are considered to survive treatment and promote tumor recurrence. Therefore, therapeutic strategies targeting GSCs have emerged as a promising approach to improving the poor prognosis of patients with GBM [6,7].

Emerging evidence indicates that CSCs possess distinct metabolic characteristics that support their stemness and survival; therefore, these metabolic dependencies represent attractive therapeutic targets [8,9,10]. Oxidative phosphorylation (OXPHOS) also plays a pivotal role in maintaining the survival and stem-like properties of GSCs. Previous studies demonstrated that the inhibition of OXPHOS activity or disruption of its regulatory mechanisms impairs GSC survival and tumorigenic potential [9,11,12].

More recently, therapeutic strategies targeting redox homeostasis, which is closely linked to mitochondrial metabolism, have attracted considerable attention [13,14]. Redox homeostasis refers to the dynamic balance between the production of reactive oxygen species (ROS) and the antioxidant defense mechanisms that neutralize them [15]. The disruption of this balance, whereby ROS production exceeds the cellular antioxidant capacity, results in excessive ROS accumulation and ultimately induces oxidative stress-mediated cell death [16]. CSCs generally maintain low intracellular ROS levels, which are considered to contribute to the maintenance of stemness and therapeutic resistance [17,18]. Similarly, in GSCs, the maintenance of a low-ROS state has been implicated in tumor progression and treatment resistance [19], whereas excessive intracellular ROS accumulation has been shown to impair GSC maintenance [19,20]. Therefore, therapeutic strategies that disrupt ROS homeostasis by enhancing ROS production or inhibiting antioxidant defense systems may selectively eliminate GSCs by inducing oxidative stress in these cells, which rely on a low-ROS state for survival [13,14,21].

Based on these findings, we focused on capsaicin and its structural analogs as candidate compounds capable of disrupting redox homeostasis in GSCs. Capsaicin, a well-characterized agonist of the transient receptor potential vanilloid 1 (TRPV1) channel, a Ca^2+^-permeable non-selective cation channel, is known to increase intracellular Ca^2+^ levels [22,23]. Since Ca^2+^ signaling is closely associated with mitochondrial function and ROS production [24], capsaicin has been reported to induce mitochondrial dysfunction, ROS generation, and subsequent cell death in various cancer cell types [25,26]. In non-stem glioma cells, capsaicin also increases intracellular Ca^2+^ levels and ROS production, ultimately triggering apoptotic cell death [22,27]. However, the effects of capsaicin and its structural analogs on GSCs remain largely unknown. Therefore, the present study investigated whether capsaicin and its structural analogs, arvanil and olvanil, which have been reported to suppress cell proliferation in several cancer models, including GBM, with low cytotoxicity toward normal cells [28,29,30], induced cell death preferentially in GSCs. We also examined the involvement of intracellular ROS and Ca^2+^ dynamics in arvanil-induced cell death.

## 2. Results

### 2.1. Arvanil and Olvanil Selectively Suppress the Viability of GSCs at Concentrations That Are Non-Toxic to Normal Cells

**Figure 1:**
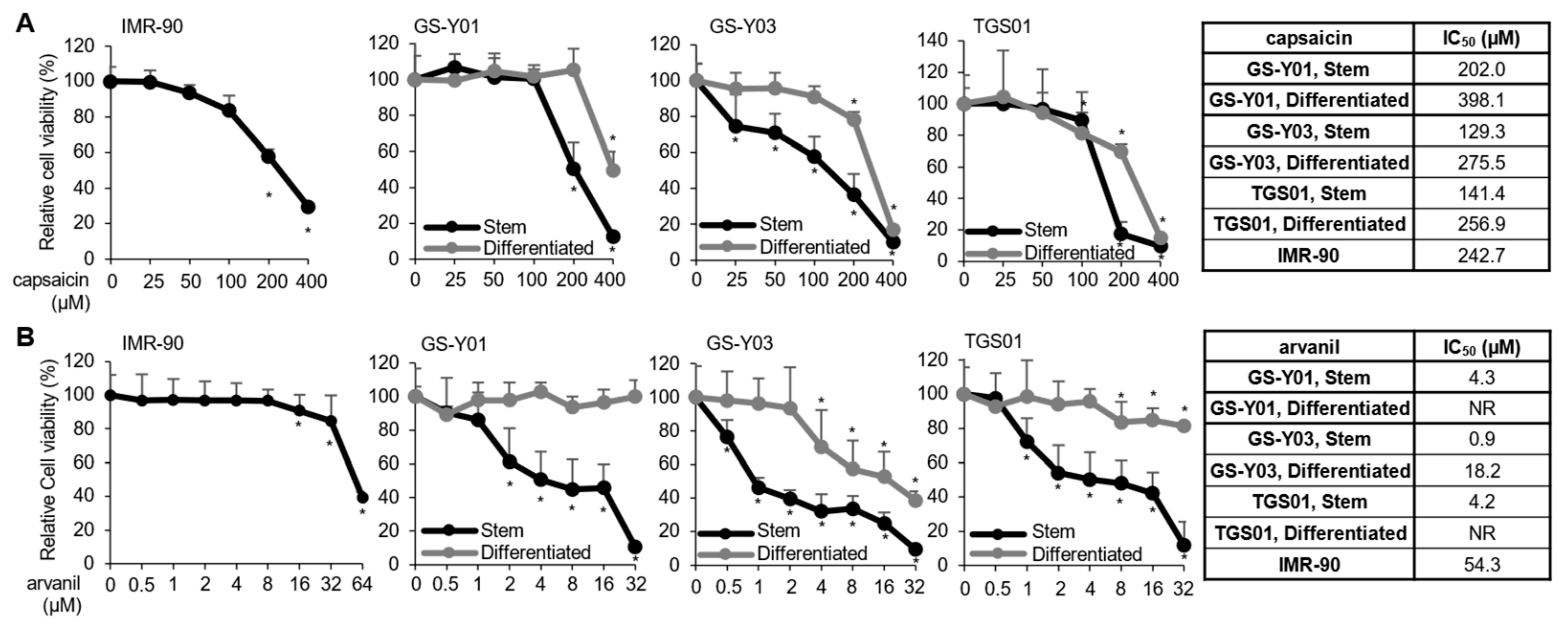
Arvanil selectively reduces glioma stem cell viability at concentrations with minimal effects on normal cell viability. (**A**) The indicated glioma stem cells (Stem: GS-Y01, GS-Y03, and TGS01), their differentiated counterparts (Differentiated), and IMR-90 cells were treated with capsaicin at the concentrations shown for 3 days and were then subjected to the WST-8 assay. (**B**) The indicated glioma stem cells (Stem: GS-Y01, GS-Y03, and TGS01), their differentiated counterparts (Differentiated), and IMR-90 cells were treated with arvanil at the concentrations shown for 3 days and were then subjected to the WST-8 assay. * *p* < 0.05 vs. the treatment without arvanil (i.e., at 0 nM). NR, not reached within the tested range. Values are presented as the means + SD of triplicate samples from a representative experiment. Similar results were obtained from two independent biological replicates.

To select the concentration range of capsaicin that is safe for use in subsequent experiments, we first examined its effects on the viability of IMR-90 human normal lung fibroblasts. Capsaicin reduced the viability of IMR-90 cells in a concentration-dependent manner, with a significant reduction being observed at concentrations of 200 μM or higher (IC_50_: 242.7 µM) (Figure 1A). We then compared the effects of capsaicin on the viability of GSCs and their genetically matched differentiated counterparts (differentiated GSCs). Capsaicin reduced cell viability more potently in GSCs than in differentiated GSCs (IC_50_: GS-Y01, Stem 202.0 µM; GS-Y01, Differentiated 398.1 µM; GS-Y03, Stem 129.3 µM; GS-Y03, Differentiated 275.5 µM; TGS01, Stem 141.4 µM; TGS01, Differentiated 256.9 µM) (Figure 1A). These results indicate that capsaicin preferentially decreased the viability of GSCs relative to differentiated GSCs. However, the concentration range at which GSC viability was effectively suppressed while preserving the viability of IMR-90 cells was narrow. Previous studies demonstrated that capsaicin is highly pungent, is known to induce pain and burning sensations, and may cause gastrointestinal adverse effects, including abdominal pain, following its oral administration or gastrointestinal exposure in humans. This pronounced irritancy and dose-dependent toxicity of capsaicin may represent major limitations to its systemic therapeutic application [31].

Therefore, we focused on the capsaicin analogs arvanil and olvanil as candidate compounds that retain capsaicin-like pharmacological activity, but with less irritancy and lower toxicity toward normal cells. Arvanil and olvanil are long-chain vanilloid compounds that act as potent TRPV1 agonists and are less irritating than capsaicin [32,33]. Moreover, both compounds have been reported to suppress tumor cell proliferation or invasion in several cancer models while exhibiting low cytotoxicity toward normal cells [28,29,30]. Based on these findings, we considered arvanil and olvanil to represent promising candidate compounds that retain capsaicin-like antitumor activity while exhibiting low irritancy and toxicity toward normal cells.

We next examined the effects of arvanil on GSC viability. To evaluate its effects on normal cells, IMR-90 cells were first treated with arvanil. Arvanil did not markedly affect IMR-90 cell viability at concentrations up to 32 μM (IC_50_: IMR-90 54.3 µM) (Figure 1B). We then investigated the effects of arvanil on the viability of GSCs and differentiated GSCs. Arvanil more strongly suppressed the viability of GSCs than that of differentiated GSCs within a concentration range that did not markedly affect the viability of normal cells (IC_50_: GS-Y01, Stem 4.3 µM; GS-Y01, Differentiated not reached within the tested range; GS-Y03, Stem 0.91 µM; GS-Y03, Differentiated 18.2 µM; TGS01, Stem 4.25 µM; TGS01, Differentiated not reached within the tested range) (Figure 1B). A similar analysis was performed with olvanil. Olvanil also more strongly suppressed the viability of GSCs than that of differentiated GSCs within a concentration range that did not markedly affect the viability of normal cells (Supplemental Figure S1). Furthermore, when selectivity indices based on the IC_50_ values for IMR-90 cells and GSCs [IC_50_ (IMR-90)/IC_50_ (GSC)] were compared, arvanil and olvanil both exhibited markedly higher selectivity for GSCs than capsaicin (Supplemental Table S1).

Collectively, these results demonstrate that arvanil and olvanil more selectively suppressed the viability of GSCs than that of differentiated GSCs at concentrations that did not affect the viability of normal cells and also exhibited selectivity over a broader concentration range than capsaicin.

### 2.2. Arvanil and Olvanil Selectively Induce Cell Death in GSCs at Concentrations That Are Non-Toxic to Normal Cells

**Figure 2:**
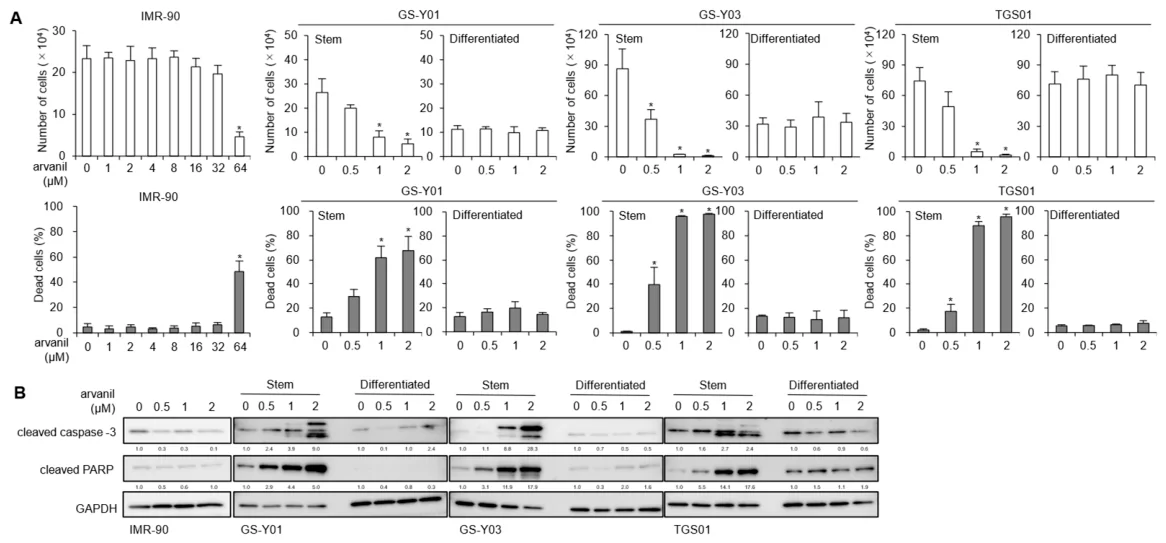
Arvanil selectively induces cell death in glioma stem cells at concentrations that do not affect normal cell viability. (**A**) The indicated glioma stem cells (Stem: GS-Y01, GS-Y03, and TGS01), their differentiated counterparts (Differentiated), and IMR-90 cells were treated for 3 days with arvanil at the indicated concentrations and were subjected to the trypan blue dye exclusion assay to measure the numbers of viable cells (upper panels) as well as the percentage of dead cells (lower panels). (**B**) The indicated glioma stem cells (Stem: GS-Y01, GS-Y03, and TGS01), their differentiated counterparts (Differentiated), and IMR-90 cells treated with the indicated concentrations of arvanil for 24 h were subjected to a Western blot analysis for the expression of the indicated proteins. The numbers below Western blot images represent relative band intensities after each band was quantified by densitometry and normalized to the GAPDH value. * *p* < 0.05 vs. cells treated without any drugs. Values represent the mean + SD of triplicate samples from a representative experiment. Similar results were obtained from two independent biological replicates.

We next evaluated the cell death-inducing effects of arvanil. Arvanil induced cell death more selectively in GSCs than in differentiated GSCs within a concentration range that did not affect the survival of normal cells (Figure 2A). Under these conditions, the treatment with arvanil preferentially induced the cleavage of caspase-3 and PARP in GSCs (Figure 2B). A similar analysis was performed with olvanil. Olvanil also induced the activation of the caspase pathway and cell death more selectively in GSCs than in differentiated GSCs within a concentration range that did not markedly affect the survival of normal cells (Supplemental Figure S2). Furthermore, since arvanil-induced cell death was almost completely suppressed by the pan-caspase inhibitor Z-VAD-FMK (Supplemental Figure S3), these results demonstrate that arvanil induced cell death in GSCs by activating the caspase pathway. Collectively, these results demonstrate that arvanil and olvanil acted as cytotoxic agents that induced cell death more selectively in GSCs than in differentiated GSCs at concentrations that did not affect the survival of normal cells.

### 2.3. Arvanil-Induced Cell Death in GSCs Is Suppressed by Ca^2+^ Chelation

**Figure 3:**
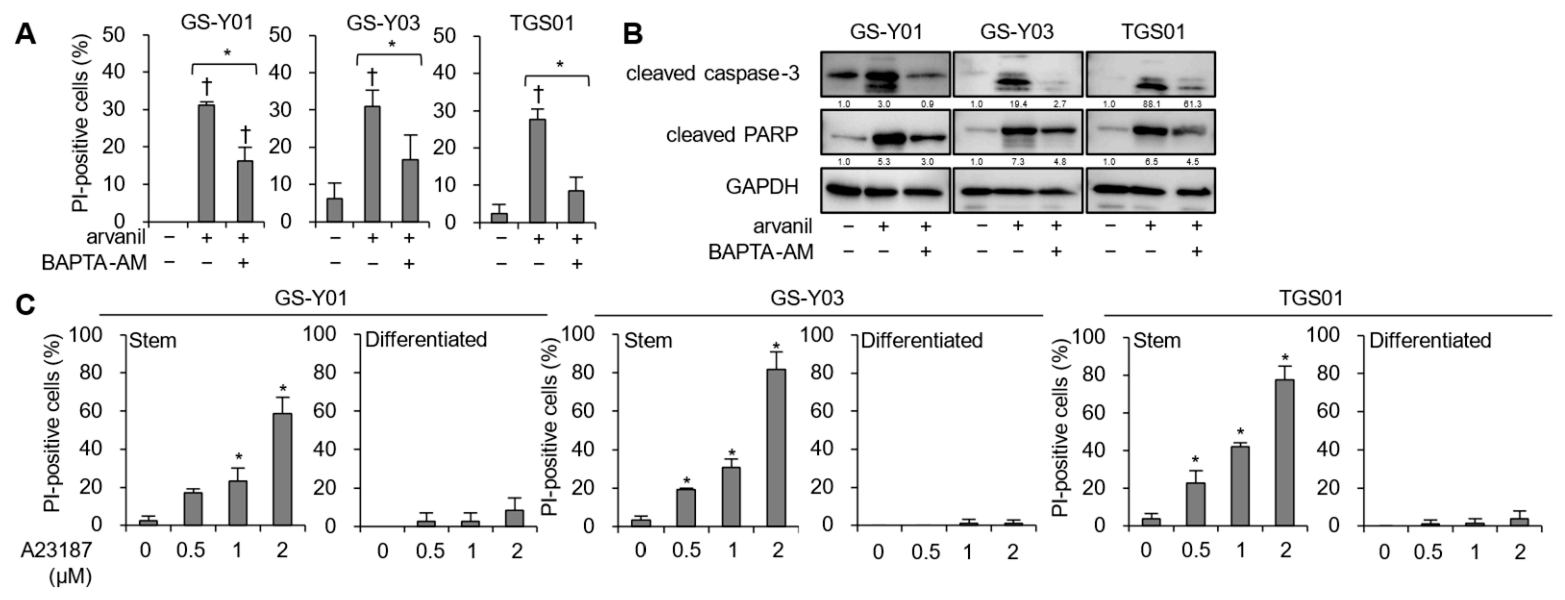
Arvanil-induced cell death in GSCs is suppressed by Ca^2+^ chelation. (**A**) The indicated glioma stem cells (GS-Y01, GS-Y03, and TGS01) were pretreated with BAPTA-AM (3 µM) for 30 min, subsequently treated with arvanil (1 µM for GS-Y03; 2 µM for GS-Y01, TGS01) for 18 h in GS-Y03, 24 h in GS-Y01, and 20 h in TGS01, and then subjected to the propidium iodide (PI) incorporation assay. Values represent the mean + SD of triplicate samples from a representative experiment. * *p* < 0.05. † *p* < 0.05 vs. cells treated without any drugs. (**B**) The indicated glioma stem cells (GS-Y01, GS-Y03, and TGS01) were treated as described in (**A**), and subjected to a Western blot analysis for the expression of the indicated proteins. The numbers below Western blot images represent relative band intensities after each band was quantified by densitometry and normalized to the GAPDH value. (**C**) The indicated glioma stem cells (Stem: GS-Y01, GS-Y03, and TGS01) and their differentiated counterparts (Differentiated) were treated with A23187 at the indicated concentration for 24 h, and were then subjected to the propidium iodide (PI) incorporation assay. Values represent the mean + SD of triplicate samples of a representative experiment. * *p* < 0.05. Similar results were obtained from two independent biological replicates.

Capsaicin is known to act as an agonist of TRPV1 and to increase intracellular Ca^2+^ levels [22,23]. Furthermore, the capsaicin-like compound arvanil has been reported to induce TRPV1-dependent Ca^2+^ signaling and subsequent cell death in high-grade astrocytomas [34]. Therefore, we investigated the involvement of intracellular Ca^2+^ in arvanil-induced cell death in GSCs. Cells were pretreated with the membrane-permeable Ca^2+^ chelator BAPTA-AM before the treatment with arvanil [35]. Arvanil-induced cell death in GSCs was significantly suppressed by the BAPTA-AM pretreatment (Figure 3A and Figure S4). In addition, arvanil-induced increases in cleaved caspase-3 and cleaved PARP were markedly attenuated by the BAPTA-AM pretreatment (Figure 3B). These inhibitory effects of BAPTA-AM suggest the involvement of intracellular Ca^2+^ in arvanil-induced cell death in GSCs. Previous studies reported that glioma-initiating cells (GICs), which exhibit a similar undifferentiated expression profile to that of neural stem cells (NSCs), were more vulnerable to the disruption of intracellular Ca^2+^ homeostasis than non-GICs [36]. Therefore, we investigated whether sensitivity to intracellular Ca^2+^ influx differed between GSCs and differentiated GSCs using the Ca^2+^ ionophore A23187. A23187 induced cell death more potently in GSCs than in differentiated GSCs (Figure 3C and Figure S4). The greater sensitivity of GSCs to A23187 was consistent with previous findings. These results suggest that GSCs were more vulnerable to the increase in intracellular Ca^2+^ levels than differentiated GSCs and also that arvanil may induce cell death by the perturbation of Ca^2+^ signaling.

### 2.4. Arvanil Preferentially Induces Intracellular ROS Production in GSCs

**Figure 4:**
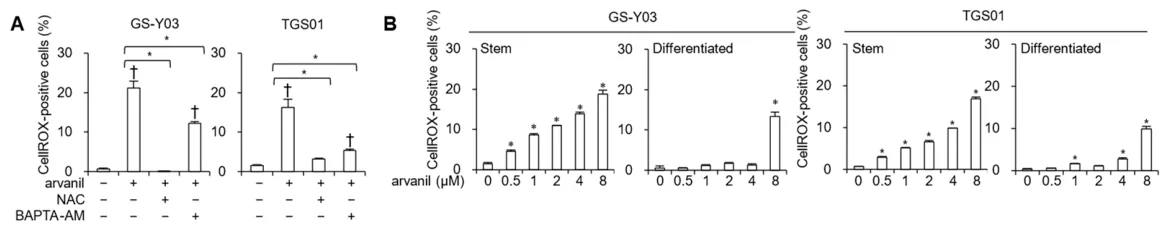
Arvanil preferentially increases oxidative stress in GSCs through Ca^2+^-related mechanisms. (**A**) The indicated glioma stem cells (GS-Y03 and TGS01) were pretreated with N-acetyl-cysteine (NAC, 10 mM) or BAPTA-AM (3 µM) for 30 min, subsequently treated with arvanil (2 µM) for 6 h, and then subjected to a CellROX assay. (**B**) The indicated glioma stem cells (Stem: GS-Y03 and TGS01) and their differentiated counterparts (Differentiated) were incubated with or without arvanil at the indicated concentrations for 6 h, and were then subjected to a CellROX assay. * *p* < 0.05. † *p* < 0.05 vs. cells treated without any drugs. Values are presented as the mean + SD of triplicate samples from a representative experiment. Similar results were obtained from two independent biological replicates.

Previous studies reported that capsaicin increased intracellular Ca^2+^ levels and induced ROS production in non-stem glioma cells, thereby leading to cell death [27]. In addition, the present results suggest the involvement of the perturbation of Ca^2+^ dynamics in arvanil-induced cell death in GSCs. Therefore, we investigated the effects of arvanil on oxidative stress in GSCs. Intracellular ROS levels were measured using CellROX Green. The arvanil treatment significantly increased intracellular ROS levels in GSCs. In contrast, the arvanil-induced increase in intracellular ROS levels was significantly suppressed by the pretreatment with the thiol antioxidant N-acetyl-L-cysteine (NAC), a potent reducing agent. Furthermore, the pretreatment with BAPTA-AM significantly suppressed the arvanil-induced increase in intracellular ROS levels (Figure 4A). These results indicate that arvanil enhanced oxidative stress in GSCs and also that intracellular Ca^2+^ may be involved in this process. Since BAPTA-AM suppressed the arvanil-induced increase in intracellular ROS levels, the perturbation of intracellular Ca^2+^ signaling may precede the oxidative stress response.

We then investigated whether the oxidative stress response induced by arvanil was associated with the difference observed in arvanil sensitivity between GSCs and differentiated GSCs. To achieve this, intracellular ROS levels were compared between the two cell types following the arvanil treatment. Arvanil increased intracellular ROS levels in a concentration-dependent manner in both cell types. However, the increase in intracellular ROS levels was observed at lower concentrations and was markedly greater in GSCs than in differentiated GSCs (Figure 4B). Collectively, these results demonstrate that arvanil enhanced intracellular oxidative stress more in GSCs than in differentiated GSCs.

### 2.5. Arvanil Induces Cell Death in GSCs Involving Mitochondria-Related Oxidative Stress

**Figure 5:**
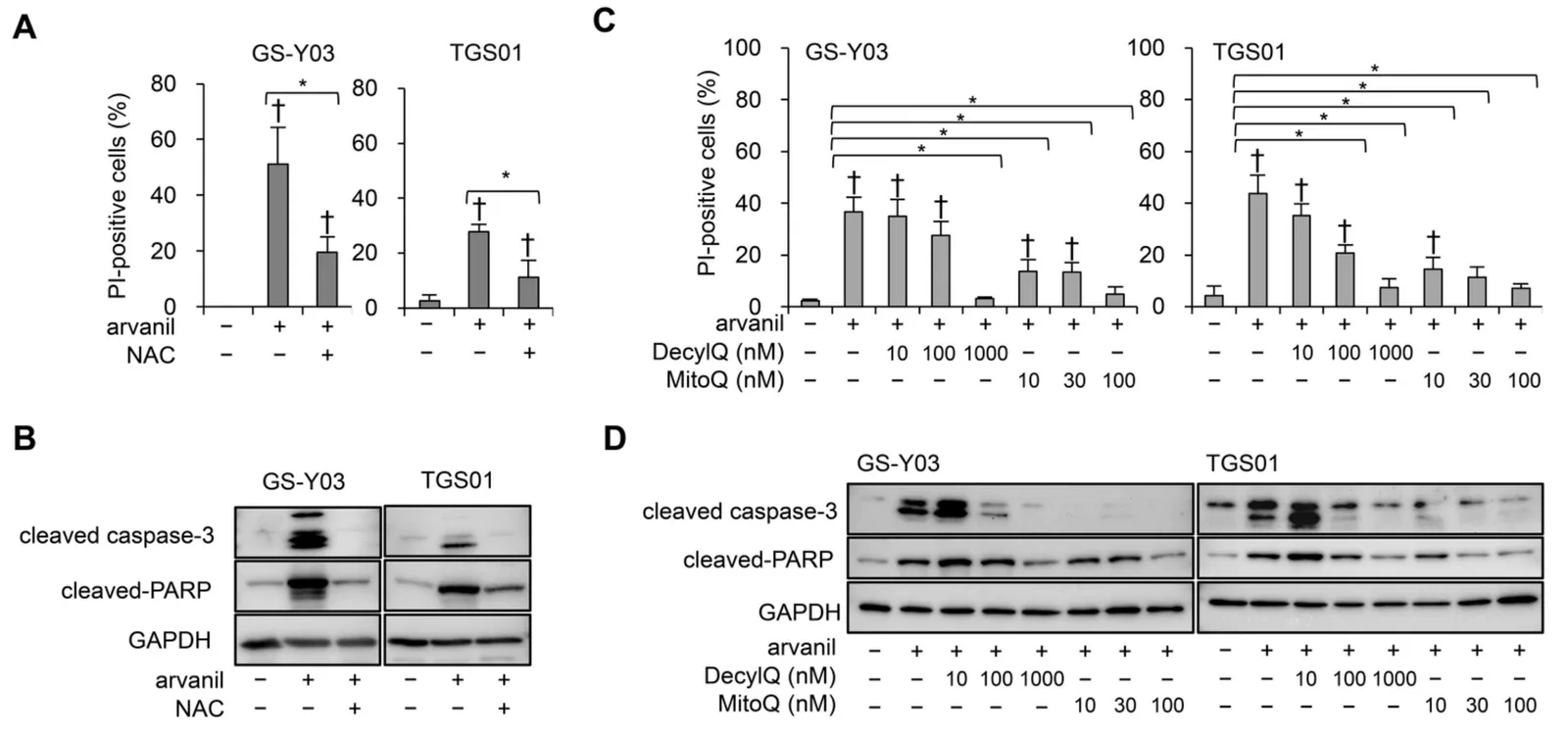
Arvanil induces cell death in GSCs through mitochondria-related oxidative stress. (**A**) The indicated glioma stem cells (GS-Y03 and TGS01) were pretreated with NAC (10 mM) for 30 min and subsequently treated with arvanil (1 µM for GS-Y03; 2 µM for TGS01) for 18 h in GS-Y03 and 20 h in TGS01, and were then subjected to the propidium iodide (PI) incorporation assay. Values represent the mean + SD of triplicate samples from a representative experiment. (**B**) The indicated glioma stem cells (GS-Y03 and TGS01) were treated as described in (**A**), and subjected to a Western blot analysis for the expression of the indicated proteins. (**C**) The indicated glioma stem cells (GS-Y03 and TGS01) were pretreated with DecylQ or MitoQ for 30 min at the concentrations indicated in the figure, subsequently treated with arvanil (1 µM for GS-Y03; 2 µM for TGS01) for 18 h in GS-Y03 and 20 h in TGS01, and then subjected to the propidium iodide (PI) incorporation assay. (**D**) The indicated glioma stem cells (GS-Y03 and TGS01) were treated as described in (**C**), and subjected to a Western blot analysis for the expression of the indicated proteins. The numbers below Western blot images represent relative band intensities after each band was quantified by densitometry and normalized to the GAPDH value. * *p* < 0.05. † *p* < 0.05 vs. cells treated without any drugs. Values represent the mean + SD of triplicate samples from a representative experiment. Similar results were obtained from two independent biological replicates.

The present results demonstrate that arvanil enhanced intracellular oxidative stress by perturbing Ca^2+^ dynamics in GSCs. In addition, the magnitude of this oxidative stress response was similar to the difference in arvanil sensitivity observed between GSCs and differentiated GSCs in the cell viability assay. Therefore, we investigated whether the arvanil-induced increase in oxidative stress functionally contributed to cell death in GSCs. The pretreatment with the antioxidant NAC significantly suppressed arvanil-induced cell death in GSCs. In addition, arvanil-induced increases in cleaved caspase-3 and cleaved PARP were suppressed by the NAC pretreatment (Figure 5A,B and Figure S5). These results suggest that the increase in oxidative stress induced by arvanil functionally contributed to GSC cell death.

We next examined the major source of ROS induced by arvanil. Mitochondria are one of the major intracellular sources of ROS, and the disruption of intracellular Ca^2+^ homeostasis has been reported to promote mitochondrial dysfunction and increase mitochondrial ROS production [37,38]. Therefore, we examined the involvement of mitochondria-related oxidative stress in arvanil-induced cell death using the mitochondria-targeted antioxidant mitoquinone mesylate (MitoQ) and, for comparison, the non-mitochondria-targeted antioxidant decylubiquinone (DecylQ) [39,40]. MitoQ and DecylQ both suppressed arvanil-induced cell death and caspase pathway activation in GSCs in a concentration-dependent manner. However, DecylQ required markedly higher concentrations than MitoQ to achieve a similar protective effect (Figure 5C,D and Figure S5). These results suggest that the selective accumulation of MitoQ in mitochondria enhanced its inhibitory effects on arvanil-induced cell death. Collectively, these results support the involvement of mitochondria-related oxidative stress in arvanil-induced cell death in GSCs.

## 3. Discussion

GSCs are a small subpopulation of cancer cells characterized by both a high degree of therapeutic resistance and tumor-initiating capacity. Since GSCs may survive apparently effective therapy and subsequently give rise to tumor recurrence, they are regarded as one of the major obstacles to the curative treatment of cancer. Therefore, the effective targeting of GSCs is considered to be important for preventing tumor recurrence and ultimately achieving long-term survival and a cure in patients with GBM [9]. In the present study, we investigated the effects of capsaicin and its structural analogs, arvanil and olvanil, on the survival of GSCs with the aim of identifying safer and more effective therapeutic agents that selectively target GSCs.

In this study, we found that capsaicin suppressed cell viability more potently in GSCs than in differentiated GSCs; however, its selectivity over normal cells was limited. In contrast, the capsaicin analogs arvanil and olvanil selectively induced cell death in GSCs at concentrations that did not markedly affect the survival of either normal cells or differentiated GSCs. Under this condition, arvanil increased the levels of cleaved caspase-3 and cleaved PARP in GSCs, and because arvanil-induced cell death was suppressed by the pan-caspase inhibitor Z-VAD-FMK, it was considered to be predominantly apoptotic; however, the possible involvement of additional mechanisms, such as ferroptosis, autophagy, or necrosis, remains unclear. Further studies will be required to fully characterize the mode of cell death induced by arvanil in GSCs. Furthermore, the arvanil-induced increase in intracellular ROS levels, the activation of the caspase pathway, and cell death were all suppressed by the pretreatment with BAPTA-AM or NAC. In addition, MitoQ exerted its protective effects at markedly lower concentrations than DecylQ. Therefore, increased mitochondria-related oxidative stress resulting from the perturbation of intracellular Ca^2+^ dynamics may be involved in arvanil-induced cell death in GSCs.

Since arvanil combines the structural features of capsaicin and anandamide, this molecule is known to act as an agonist of TRPV1, a Ca^2+^-permeable non-selective cation channel, as well as cannabinoid receptor 1 (CB1), a G protein-coupled receptor, and modulates Ca^2+^ signaling in several cell types, including GBM [28,30,32,34,39]. From this perspective, we examined the effects of a combined treatment with capsaicin and anandamide on GSC viability using Synergy Finder. The combination of capsaicin and anandamide synergistically reduced GSC viability (mean Synergy score (Zero Interaction Potency (ZIP) model): GS-Y01 12.89; GS-Y03 16.45; TGS01 10.18) (Supplemental Figure S6). These results suggest that, in addition to TRPV1-mediated effects, CB1-mediated effects contributed to the synergistic reduction in cell viability. However, since arvanil has also been reported to induce cell death independently of TRPV1 and CB1 receptors [41], further studies are required to clarify the relationship between the direct molecular targets of arvanil and intracellular Ca^2+^ dynamics.

Ca^2+^ signaling has been reported to play a role in various cellular functions, including proliferation, survival, migration, and cell death, in many types of cancer cells [42]. In some CSC models, Ca^2+^ signaling pathways have also been shown to contribute to the maintenance of stemness and cell survival, and their functional inhibition may induce cell death [43]. In a comparative analysis of GICs with an undifferentiated transcriptomic profile resembling that of NSCs and GICs exhibiting more distinct characteristics from those of NSCs, the former expressed higher levels of ion channel genes associated with Ca^2+^ influx and Ca^2+^ signaling. Furthermore, these more undifferentiated GICs showed greater sensitivity to the Ca^2+^ ionophore A23187, which induces uncontrolled Ca^2+^ influx across the plasma membrane [36]. In the present study, A23187 also induced cell death more selectively in GSCs than in differentiated GSCs. These results appear to support sensitivity to the disruption of intracellular Ca^2+^ homeostasis representing a therapeutic vulnerability of GSCs.

In the present study, the arvanil-induced increase in intracellular ROS levels was observed at lower concentrations in GSCs than in differentiated GSCs and was also more pronounced in GSCs. Several GSC models, including ours, have been reported to exhibit a greater dependence on mitochondrial metabolism and OXPHOS than differentiated GSCs, and the maintenance of these metabolic processes contributes to their self-renewal and survival [9,11,12]. Mitochondria are a major source of ROS in cells. OXPHOS has been shown to promote ROS production through electron leakage from the electron transport chain. In addition, the maintenance of redox homeostasis is important for GSC survival [13,21,44]. Therefore, targeting redox homeostasis may represent a strategy for exploiting the vulnerabilities of GSCs. Arvanil, which preferentially induces oxidative stress in GSCs, may provide one example of a drug with such activity. Furthermore, in the present study, the mitochondria-targeted antioxidant MitoQ suppressed arvanil-induced cell death even at low concentrations, suggesting that arvanil induced cell death by increasing mitochondria-related oxidative stress more strongly in GSCs than in differentiated GSCs. The mechanisms by which arvanil increased mitochondria-related oxidative stress in GSCs may involve mitochondrial Ca^2+^ uptake associated with changes in cytosolic Ca^2+^ dynamics. The mitochondrial calcium uniporter (MCU) complex is known to constitute the major pathway responsible for Ca^2+^ uptake across the inner mitochondrial membrane [45]. The expression of MCU was previously shown to be higher in GSCs than in their corresponding non-stem tumor cells, and MCU-mediated mitochondrial Ca^2+^ uptake contributed to the maintenance of GSC metabolism, self-renewal capacity, and tumorigenic potential [46]. On the other hand, the dysregulation of Ca^2+^ uptake through the MCU complex may lead to the mitochondrial accumulation of Ca^2+^, increased mitochondrial ROS production, and enhanced susceptibility to cell death [47]. Furthermore, a study using hepatocellular carcinoma cells suggested that arvanil may directly bind to MICU1, a regulator of the MCU complex, and induce cell death by increasing mitochondrial Ca^2+^ uptake and ROS production [48]. Based on these findings, changes in mitochondrial Ca^2+^ uptake through the MCU complex may represent one mechanism underlying the arvanil-induced increase in Ca^2+^-dependent oxidative stress. However, the present study did not directly evaluate the molecular targets of arvanil, mitochondrial Ca^2+^ dynamics, or the functional involvement of the MCU complex; therefore, further studies are required to investigate these mechanisms.

Although the present study demonstrated the potential of arvanil as a compound capable of selectively killing GSCs in vitro at concentrations that have limited effects on the survival of normal cells and differentiated GSCs, further investigations are required before its clinical application. Future studies are needed to evaluate the safety of arvanil, its therapeutic efficacy in targeting GSCs, and its distribution in the brain and tumor tissues in in vivo mouse models. Furthermore, the effective treatment of GBM requires the simultaneous control of not only GSCs, but also non-stem glioma cells, which constitute the majority of the tumor. Therefore, an important future challenge will be to identify an optimal therapeutic strategy that effectively controls both cell populations, including combination therapy with existing treatments that are effective against non-stem glioma cells.

## 4. Materials and Methods

### 4.1. Antibodies and Reagents

Antibodies against cleaved caspase-3 (#9661), cleaved PARP (#9541), and GAPDH (#5174) were purchased from Cell Signaling Technology, Inc. (Beverly, MA, USA). Arachidonoyl ethanolamide (anandamide, 90050), arvanil (90052), and capsaicin (92350) were purchased from Cayman Chemical Company (Ann Arbor, MI, USA). Z-VAD-FMK (3188-v) was supplied by Peptide Institute Inc. (Osaka, Japan). A23187 (100105), NAC (A9165), and DecylQ (D7911) were purchased from Merck KGaA (Darmstadt, Germany). BAPTA-AM (S7534) and MitoQ (S8978) were purchased from Selleck Chemicals (Houston, TX, USA). Olvanil (HY-101323) was purchased from MedChemExpress (Monmouth Junction, NJ, USA).

### 4.2. Cell Culture

The isolation and establishment of patient-derived GSCs (GS-Y01, GS-Y03, and TGS01) were conducted as previously described [49,50,51,52]. TGS01 was kindly provided by Dr. T. Todo (University of Tokyo, Japan) [53]. GSCs were maintained under previously reported monolayer stem cell culture conditions [50,54]. The differentiation of GSCs was induced by culturing cells in DMEM/F-12 medium supplemented with 10% fetal bovine serum (FBS, Thermo Fisher Scientific Inc., Waltham, MA, USA), 100 units/mL of penicillin, and 100 μg/mL of streptomycin for 7–14 days [54]. IMR-90 (JCRN9054), a human normal fetal lung fibroblast cell line, was purchased from the Japanese Collection of Research Bioresources Cell Bank (Osaka, Japan) and maintained in DMEM supplemented with 10% FBS. All IMR-90 experiments were performed using cells with a low passage number (<8).

### 4.3. WST-8 Cell Viability Assay

Cell viability was evaluated using the WST-8 assay [12]. Cells plated in a 96-well plate (0.5–1 × 10^4^/well) were treated as described in the figure legends. WST-8 reagent (Cell Counting Kit-8, DOJINDO Laboratories, Kumamoto, Japan) was then added and cells were incubated at 37 °C for 1–3 h. Absorbance at 450 nm was measured using a microplate reader (iMark; Bio-Rad Laboratories, Inc., Hercules, CA, USA). Relative cell viability was calculated as a percentage of the absorbance of treated samples relative to that of controls. The synergistic effects of the drug combinations were evaluated using the ZIP model in the SynergyFinder 3.0 web application (https://synergyfinder.org/#!/; accessed on 4 August 2026) [55,56].

### 4.4. Trypan Blue Dye Exclusion Assay

The numbers of viable and dead cells were assessed using the trypan blue dye exclusion assay [12,52]. Cells treated with drugs as described were pipetted (GSCs) or trypsinized (differentiated GSCs and IMR-90) and suspended in phosphate-buffered saline (PBS). Cells were then stained with 0.2% trypan blue solution (Merck) for 1 min. Viable and dead cells were identified by their ability and inability, respectively, to exclude trypan blue using a hemocytometer. The assay was repeated at least twice with similar results, and one set of representative data is presented.

### 4.5. Propidium Iodide (PI) Incorporation Assay

The PI incorporation assay was performed to assess the percentage of dead cells as previously described [12,52]. Cells were incubated with PI (1 μg/mL, Thermo Fisher Scientific) and Hoechst33342 (10 μg/mL, Thermo Fisher Scientific) at 37 °C for 5 min. To calculate the ratio of PI-positive cells (dead cells) to Hoechst-positive cells (total cells), fluorescent images were obtained using a fluorescence microscope (CKX53; EVIDENT, Tokyo, Japan) equipped with an iPhone 7 (Apple Inc., Cupertino, CA, USA) and scored. More than three areas of cells were counted to calculate the percentage of PI-positive cells.

### 4.6. Western Blot Analysis

A Western blot analysis was conducted as previously described [12,52]. Cells were collected and washed with ice-cold PBS and lysed in RIPA buffer (10 mM Tris-HCl (pH 7.4), 0.1% sodium dodecyl sulfate (SDS), 0.1% sodium deoxycholate, 1% Nonidet P-40, 150 mM NaCl, 10 mM sodium fluoride, 10 mM sodium pyrophosphate, 1.5 mM sodium orthovanadate, 1 mM EDTA, and protease inhibitor cocktail set III (FUJIFILM Wako Pure Chemical Corporation, Osaka, Japan)). Lysates were immediately mixed with the same volume of 2 × Laemmli buffer (125 mM Tris-HCl (pH 6.8), 4% SDS, and 10% glycerol) and boiled at 95 °C for 10 min. The protein concentrations of the cell lysates were measured using a BCA protein assay kit (Thermo Fisher Scientific). Samples containing equal amounts of protein were separated by SDS-polyacrylamide gel electrophoresis and transferred to polyvinylidene difluoride membranes. The membranes were probed with the indicated primary antibodies followed by appropriate horseradish peroxidase (HRP)-conjugated secondary antibodies (Jackson ImmunoResearch Inc., West Grove, PA, USA), as recommended by the manufacturer of each antibody. To reprobe immunoblots, antibodies were stripped from the probed membranes using stripping buffer (2% SDS, 100 mM β-mercaptoethanol, and 62.5 mM Tris-HCl (pH 6.8)). After stripping, the membranes were washed with Tris-buffered saline with Tween 20, blocked with skim milk, and reprobed with appropriate antibodies. Immunoreactive bands were visualized using Immobilon Western Chemiluminescent HRP Substrate (Merck) and detected by a ChemiDoc Touch device (Bio-Rad Laboratories, Inc., Hercules, CA, USA). The analysis was repeated at least twice with similar results, and one set of representative data is presented. The quantification of bands on the membranes was performed by densitometry using ImageJ software (version 1.54g) (https://imagej.net/ij/, accessed on 14 September 2026).

### 4.7. Fluorescence-Activated Cell Sorting (FACS) Analysis

A FACS analysis was conducted as previously described [57]. Cells were incubated in culture medium containing 2.5 μM CellROX Green (Thermo Fischer Scientific) at 37 °C for 60 min. After being washed with PBS twice, cells were fixed in 4% paraformaldehyde at RT for 10 min, resuspended in PBS, and examined using FACSCanto II (BD Biosciences, Franklin Lakes, NJ, USA). The data obtained were analyzed using FlowJo software, version 7.6.5 (Tree Star Inc., Ashland, OR, USA).

### 4.8. Data Reproducibility and Statistical Analysis

Data analyses were performed using EZR (Version 1.70, Saitama Medical Center, Jichi Medical University), a graphical user interface for R [58], and results are expressed as the mean + standard deviation of at least triplicate samples. Additionally, the significance of differences was assessed using a one-way ANOVA followed by Tukey’s post hoc test. *p* values < 0.05 were considered to be significant and are indicated with asterisks or † in the figures. All cell experiments were performed using at least two biological replicates, with similar results, and one representative dataset is presented.

## 5. Conclusions

The present study demonstrated that arvanil and olvanil selectively killed GSCs at concentrations that exerted limited effects on the survival of normal cells and differentiated GSCs. Furthermore, arvanil preferentially induced GSC death in association with intracellular Ca^2+^ dysregulation and mitochondria-related oxidative stress, underscoring its potential as a novel therapeutic candidate that exploits GSC-specific vulnerabilities. If its efficacy and safety are demonstrated in vivo in future studies, arvanil may become a promising therapeutic candidate for GBM, which remains difficult to treat.

## Data Availability

All data are contained in this article and there are no repository data.

## Supplementary Materials

The following supporting information can be downloaded at https://www.mdpi.com/article/10.3390/ijms27209358/s1.
