# Supplementary material for: Targeting Redox Homeostasis with Arvanil Is Preferentially Cytotoxic to Glioma Stem Cells
Source: Int J Mol Sci. 2026 Oct 21;27(20):9358. doi: 10.3390/ijms27209358 (PMC13705373; doi:10.3390/ijms27209358)
Supplement: Supplementary file 1 [file ijms-27-09358-s001.zip › ijms-4553701-supplementary.pdf]

Supplemental Figure S1

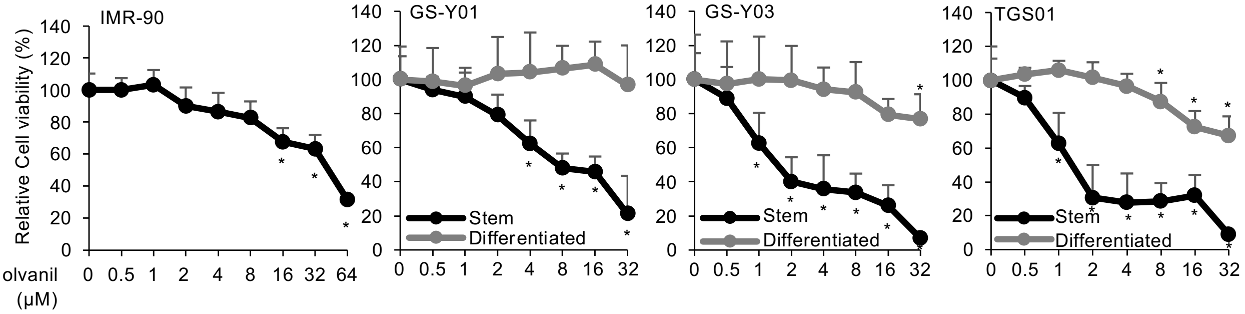

olvanil	IC_{50} (μM)
GS-Y01, Stem	7.2
GS-Y01, Differentiated	NR
GS-Y03, Stem	1.5
GS-Y03, Differentiated	NR
TGS01, Stem	1.3
TGS01, Differentiated	NR
IMR-90	42.6

Supplemental Figure S1. Olvanil selectively reduces glioma stem cell viability at concentrations with minimal effects on normal cell viability. The indicated glioma stem cells (Stem: GS-Y01, GS-Y03, and TGS01), their differentiated counterparts (Differentiated), and IMR-90 cells were treated with olvanil at the concentrations shown for 3 days and were then subjected to the WST-8 assay. * $p < 0.05$ vs. the treatment without olvanil (i.e., at 0 nM). NR, not reached within the tested range.

Supplemental Table S1

Selectivity Index (SI)	capsaicin	arvanil	olvanil
IMR-90/GS-Y01 Stem	1.2	12.6	5.91
IMR-90/GS-Y03 Stem	1.8	59.7	28
IMR-90/TGS01 Stem	1.7	35.7	30.6

*Selectivity Index (SI) =IC_{50} (IMR-90) / IC_{50} (GSC)

Supplemental Table S1. The selectivity index (SI) was calculated by dividing the IC50 value derived from the WST-8 cell viability assay for IMR-90 cells by that for each glioma stem cell (GS-Y01, GS-Y03, and TGS01). A higher SI indicates greater selectivity for GSCs over IMR-90 cells.

Supplemental Figure S2

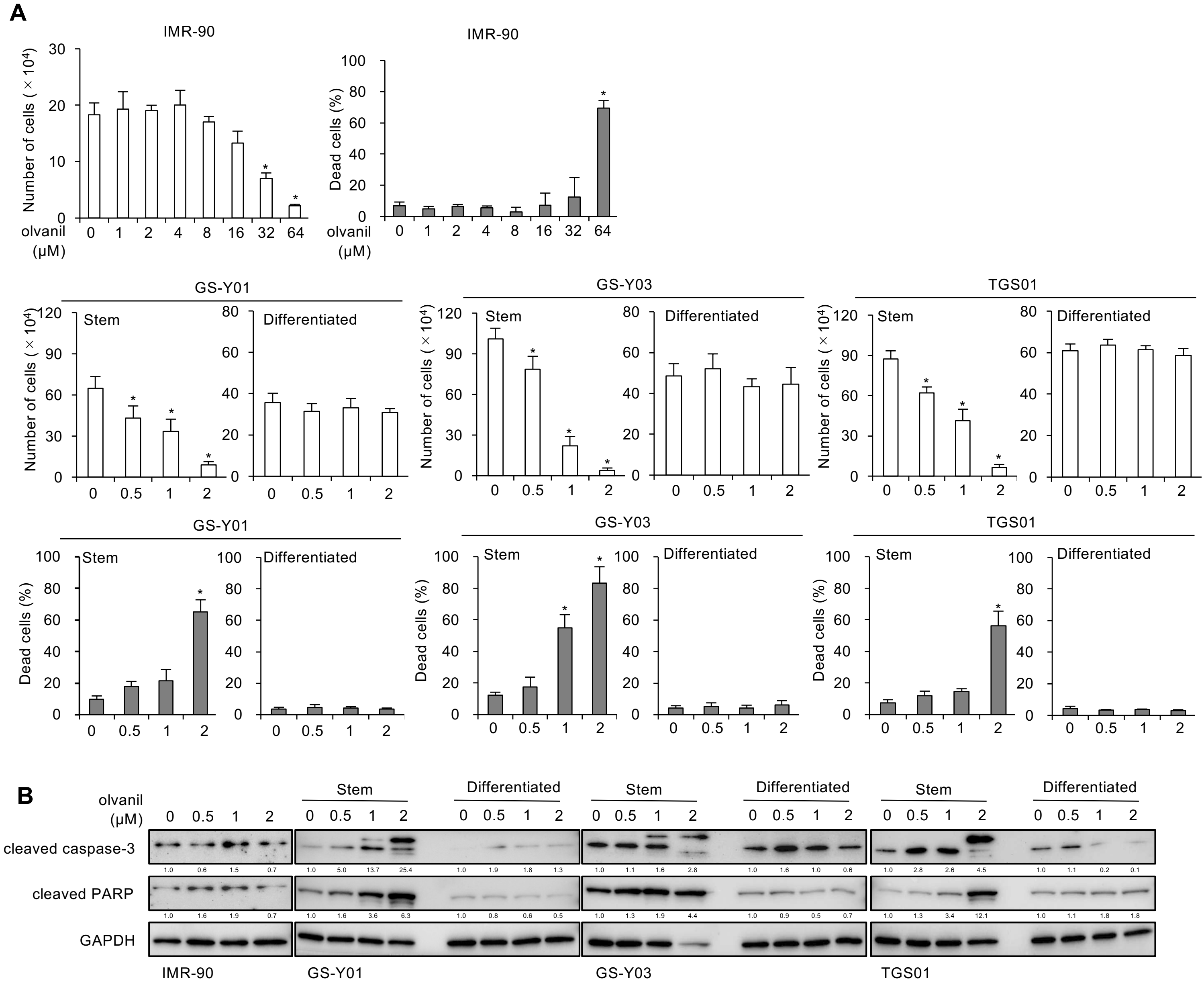

Supplemental Figure S2. Olvanil selectively induces cell death in glioma stem cells at concentrations that do not affect normal cell viability. (A) The indicated glioma stem cells (Stem: GS-Y01, GS-Y03, and TGS01), their differentiated counterparts (Differentiated), and IMR-90 cells were treated for 3 days with olvanil at the indicated concentrations and were subjected to the trypan blue dye exclusion assay to measure the number of viable cells as well as the percentage of dead cells (lower panels). Values represent the mean + SD of triplicate samples of a representative experiment. * $p < 0.05$ vs. cells treated without any drugs. (B) The indicated glioma stem cells (Stem: GS-Y01, GS-Y03, and TGS01), their differentiated counterparts (Differentiated), and IMR-90 cells treated with the indicated concentrations of olvanil for 3 days were subjected to a Western blot analysis for the expression of the indicated proteins. The numbers below Western blot images represent relative band intensities after each band was quantified by densitometry and normalized to the GAPDH value.

Supplemental Figure S3

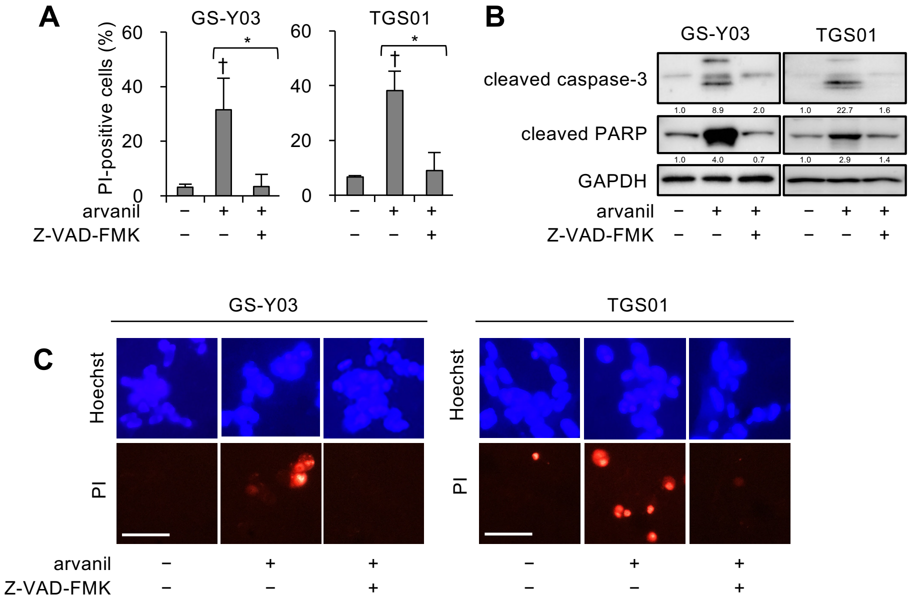

Supplemental Figure S3. Arvanil-induced cell death is suppressed by Z-VAD-FMK. (A) The indicated glioma stem cells (GS-Y03 and TGS01) were pretreated with Z-VAD-FMK (40 μM) for 30 min, subsequently treated with arvanil (1 μM for GS-Y03; 2 μM for TGS01) for 24 h, and then subjected to the propidium iodide (PI) incorporation assay. Values represent the mean + SD of triplicate samples of a representative experiment. * $p < 0.05$. † $p < 0.05$ vs. cells treated without any drugs. (B) The indicated glioma stem cells (GS-Y03 and TGS01) were treated as described in (A), and subjected to a Western blot analysis for the expression of the indicated proteins. The numbers below Western blot images represent relative band intensities after each band was quantified by densitometry and normalized to the GAPDH value. (C) Representative images are shown. Bars; 50 μm.

Supplemental Figure S4

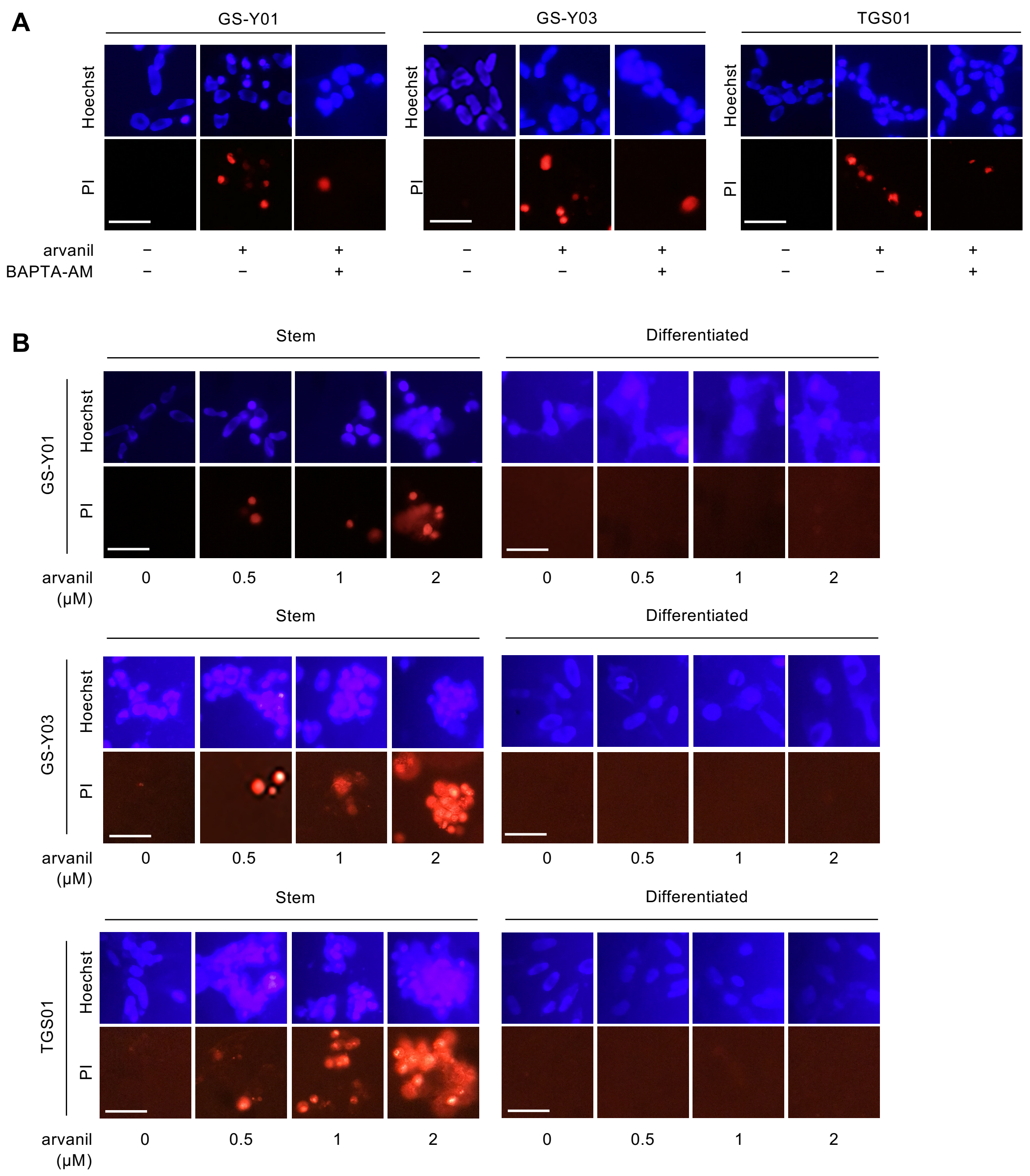

Supplemental Figure S4. Representative Images. (A) Representative images corresponding to Figure 3A. Bars; 50 µm. (B) Representative images corresponding to Figure 3C. Bars; 50 µm.

Supplemental Figure S5

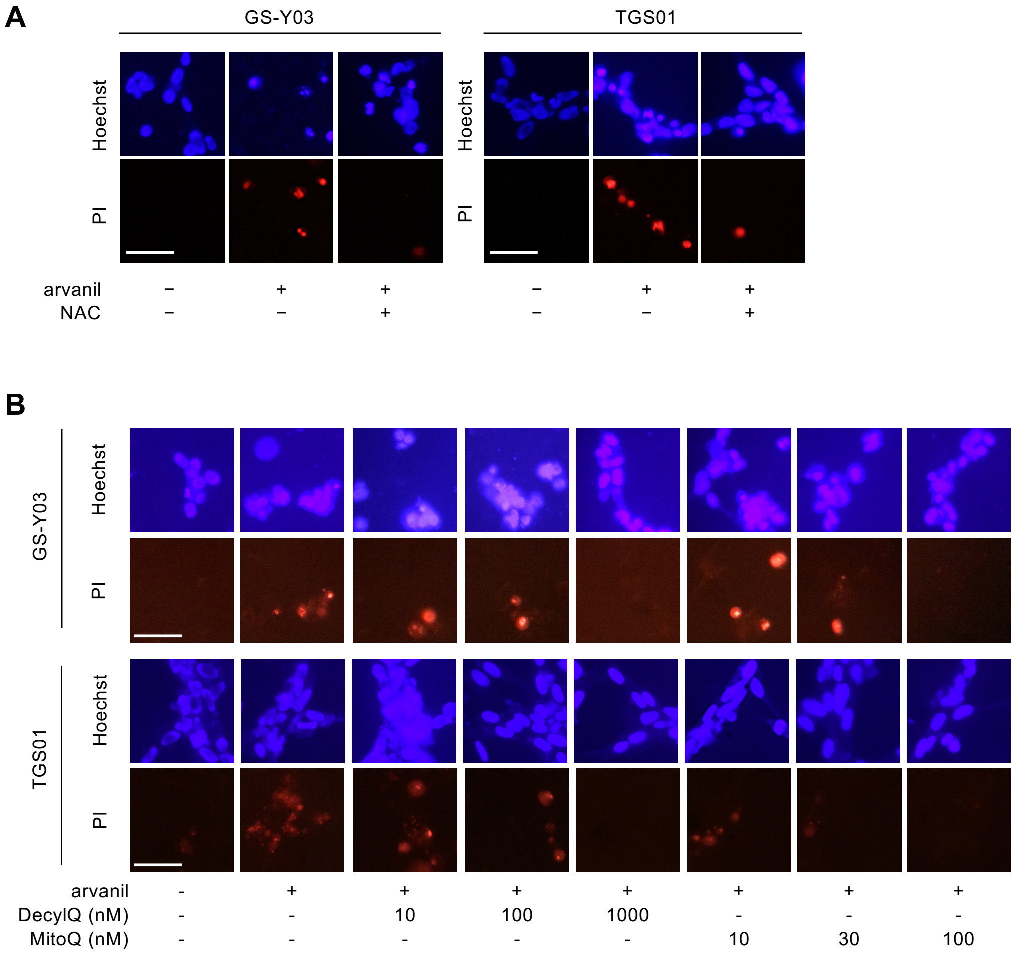

Supplemental Figure S5. Representative Images. (A) Representative images corresponding to Figure 5A. (B) Representative images corresponding to Figure 5C. Bars; 50 µm.

Supplemental Figure S6

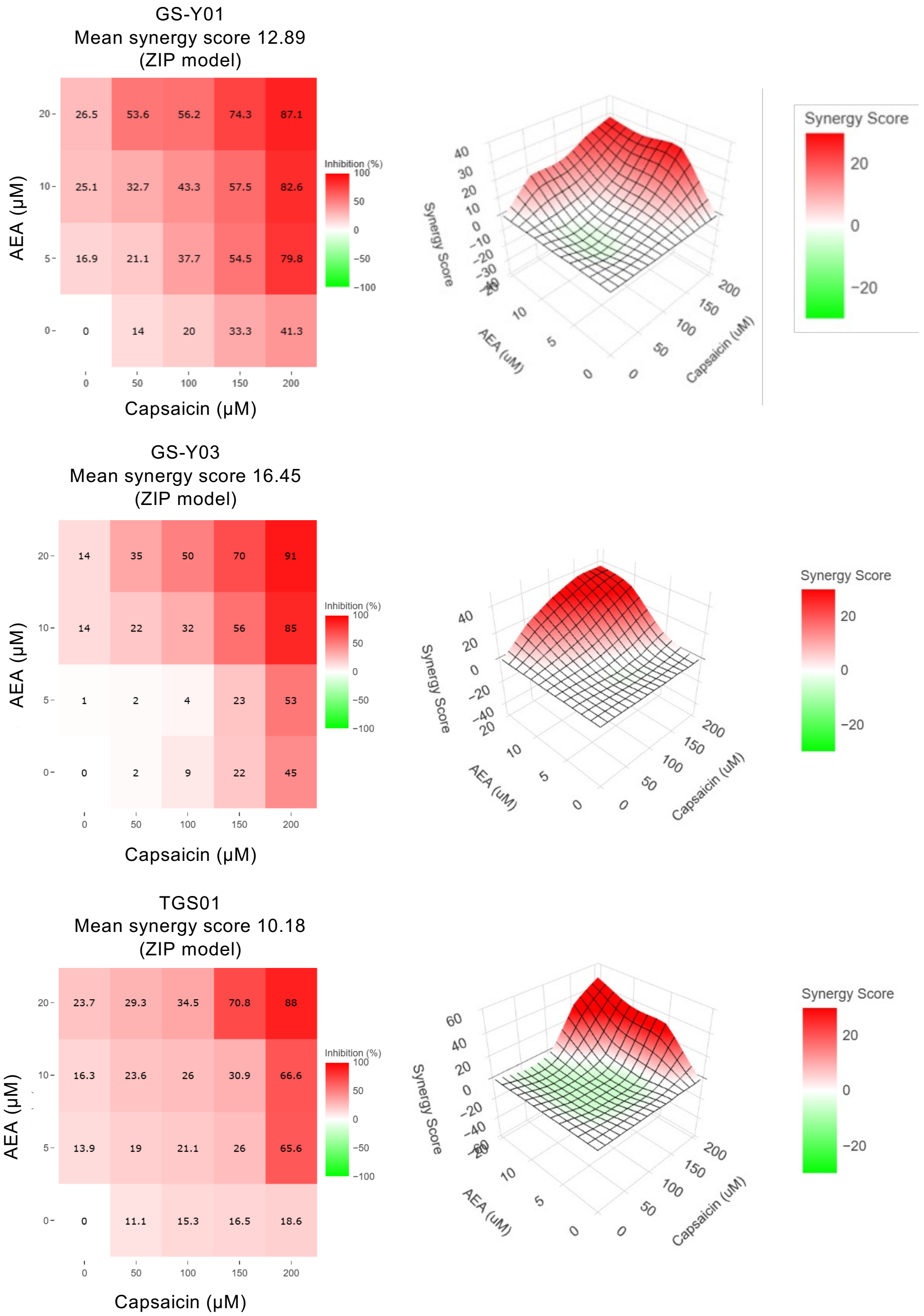

Supplemental Figure S6. Capsaicin and anandamide synergistically inhibit GSC proliferation. The combination was evaluated by the WST-8 assay after 3 days in glioma stem cells (GS-Y01, GS-Y03, and TGS01). Synergy Finder software (synergyfinder 3.0) (https://synergyfinder.org/#!/) was used to evaluate synergistic effects by the Zero Interaction Potency (ZIP) model. The percentages of cell growth inhibition relative to the Control (capsaicin–, anandamide (AEA)–) are indicated in each square and shown as heat maps. The synergy score for each combination is shown above each image.
